# Silent Spread of *Borrelia* Infection in Sardinia, Italy: Implications for Integrated Surveillance in the Mediterranean

**DOI:** 10.3390/healthcare13212709

**Published:** 2025-10-27

**Authors:** Antonella Arghittu, Grazia Galleri, Laura Mameli, Roberto Manetti, Mark J. Soloski, Ivana Piredda, Giovanna Deiana, Alessandra Palmieri, Marco Dettori, Giuseppe Satta, Paolo Castiglia

**Affiliations:** 1 Department of Medicine, Surgery and Pharmacy, University of Sassari, 07100 Sassari, Italy; aarghittu@uniss.it (A.A.); rmanetti@uniss.it (R.M.); castigli@uniss.it (P.C.); 2 Department of Biomedical Sciences, University of Sassari, 07100 Sassari, Italy; galleri@uniss.it (G.G.); lauramameli.lm@gmail.com (L.M.); 3 University Hospital of Sassari, 07100 Sassari, Italy; giovanna.deiana90@gmail.com (G.D.); luca@uniss.it (A.P.); 4 Lyme Disease Research Center, Division of Rheumatology, Department of Medicine, Johns Hopkins School of Medicine, Baltimore, MD 21224, USA; mski@jhmi.edu; 5 Department of Animal Health, Zoo-Prophylactic Institute of Sassari, 07100 Sassari, Italy; ivana.piredda@izs-sardegna.it (I.P.); giuseppe.satta@izs-sardegna.it (G.S.)

**Keywords:** *Borrelia* spp., seroprevalence, infection, Lyme borreliosis, One Health strategies, endemicity signals

## Abstract

**Background**: Lyme borreliosis (LB) constitutes a major challenge for Public Health, particularly in regions where surveillance and diagnostic systems are underdeveloped or fragmented. Despite its potential as a hotspot for tick-borne diseases, Sardinia (Italy) remains poorly explored in terms of LB epidemiology. **Methods**: A sero-prevalence study was conducted on serum samples stored in the biobank of a hospital in Northern Sardinia. The serum library consisted of serum samples collected on the basis of a diagnostic hypothesis of rheumatic disease. Serological testing for antibodies against *Borrelia* was performed using the indirect immunofluorescence assay (IFA) and enzyme-linked immunosorbent assays (ELISA), followed by confirmation by Western blot for positive results. The study analyzed 58 serum samples from patients selected based on clinical symptoms compatible with *Borrelia* spp. infection. **Results**: Among the 58 patients, 9 (15.5%) yielded positive results, with absorbance values higher than those of the positive control, suggesting that the pathogen is widespread but poorly recognized in Sardinia. The results are in line with broader trends in the Mediterranean, indicating that Sardinia can no longer be considered a marginal area for *Borrelia* spp. circulation. **Conclusions**: The status of Sardinia as a sentinel territory underlines the need for enhanced epidemiological surveillance within the One Health approach, including human, animal and environmental health.

## 1. Introduction

Lyme borreliosis (LB) is a multisystemic infection caused by spirochetes of the genus *Borrelia*. It is the most prevalent tick-borne zoonosis in the Northern Hemisphere and its distribution is predominantly circumscribed to temperate regions of North America, Europe, and parts of Asia [1,2,3,4,5,6].

It is transmitted to humans chiefly through the bite of an infected tick, primarily *Ixodes ricinus* in Europe, while *Ixodes scapularis* and *Ixodes pacificus* serve as the main vectors in North America. Other ixodid species, such as *Ixodes gibbosus*, may act as secondary vectors [7,8]. The remarkable ecological plasticity of *Ixodes ricinus*, capable of parasitizing over 237 vertebrate species, enables it to survive in diverse biotopes, as deciduous and mixed forests characterized by high humidity and abundant underbrush [2,7,9].

LB epidemiology relies on diverse reservoirs (small rodents, cervids, lagomorphs, insectivores, reptiles), with passerines driving pathogen spread along migratory routes [10]. Humans and domestic animals are instead considered accidental hosts, without any role in maintaining the enzootic cycle [9,10,11,12].

Clinically, LB presents with a polymorphic spectrum of clinical manifestations that makes it difficult to recognize in a timely manner [13]. The early symptoms, erythema migrans, may go unnoticed or be misinterpreted [14]. This diagnostic uncertainty is particularly common in settings with low awareness, but may also occur in endemic regions, where atypical or transient presentations can still lead to under-recognition by patients or misdiagnosis [13,14].

According to recent evidence in the literature, up to 50% of infections remain asymptomatic [15], while a proportion progress to neurological, rheumatological, or cardiac complications. Among these, neuroborreliosis constitutes the most severe manifestation [8,9,10,11,12,13].

Current guidelines for the diagnostic ascertainment of *Borrelia burgdorferi* infection recommend a standard two-tiered approach, with initial screening by indirect immunofluorescence assay (IFA) or enzyme-linked immunosorbent assay (ELISA), followed by confirmation with a more specific immunoblot (Western blot) if the first test is positive or equivocal [16,17,18]. This approach is endorsed by contemporary clinical guidelines (IDSA/AAN/ACR, 2020) and remains the cornerstone of diagnostic algorithms, although alternative or modified two-tier strategies have been evaluated in recent studies and may be considered according to regional practice and test performance characteristics [16]. However, interpretation of the results remains challenging, especially for the heterogeneous antigenic profile of *Borrelia* spp. and in low-endemicity settings [18].

Given their repeated exposure to environments compatible with the ecological requirements of *Ixodes ricinus*, workers employed in forestry, agriculture, environmental monitoring, and wildlife management are plausibly at increased risk of encountering ticks and, consequently, of contracting *Borrelia* infection [19,20,21,22].

On a global scale, the disease exhibits high incidence rates in the northeastern and northwestern, midwestern United States (e.g., Massachusetts, Connecticut, Minnesota, Oregon, Wisconsin, Rhode Island, New Hampshire), as well as certain Asian regions, notably China and Hokkaido, Japan [9,23]. In Europe, an estimated 235,000 cases of LB are recorded annually. The highest incidence rates are observed in Slovenia and Austria, reporting approximately 130 and 120 cases per 100,000 inhabitants, respectively [7,24]. However, significant heterogeneity in surveillance systems, coupled with diagnostic discrepancies, severely limits interregional comparability and supports the hypothesis that it is substantially underreported [25,26].

The geographical distribution of LB is governed by the eco-climatic constraints affecting tick survival and activity, namely temperatures ranging from −10 °C to +35 °C, relative humidity above 80%, and the presence of suitable hosts Although precise epidemiological data on seasonal incidence remain scarce, the activity of *Ixodes ricinus* is known to peak during the warmer months in temperate regions, typically between late spring and summer [27,28,29]. However, ongoing climate change and alterations in the anthropogenic landscape have begun to redefine its phenology and spatial distribution, extending the high-risk period into spring and autumn. These environmental shifts are also expected to favor the gradual expansion of the tick’s geographical range, potentially leading to an increased incidence of Lyme borreliosis in previously low-risk areas. This seasonal pattern suggests that human exposure to infected ticks may similarly vary throughout the year, an aspect that warrants further investigation through structured epidemiological monitoring [29,30].

In Italy, LB was first documented in Liguria in 1985, followed by increasing reports from Friuli Venezia Giulia and Trentino-Alto Adige [31,32,33,34,35]. Over the past decade, 1260 cases have been confirmed in clinical centres, largely concentrated in Alpine regions, particularly in the Northeast [36]. Since these findings are predominantly hospital-based, they likely represent only a fraction of the true disease burden. In contrast, reports from central and southern Italy remain sporadic, while data from insular territories, Sardinia and Sicily, are markedly deficient [37,38,39].

Sardinia, with its extensive forest cover and a considerable workforce engaged in environmental and agricultural sectors, constitutes an ecosystem of strategic interest for LB surveillance. Despite the prevailing Mediterranean climate, which is traditionally deemed less conducive to the establishment of *Ixodes ricinus*, isolated entomological findings have confirmed its presence on the island [34,35,36,37]. However, the absence of a structured surveillance framework has hindered a reliable assessment of the pathogen’s actual circulation. The only sero-epidemiological study to date, conducted in the early 1990s on a cohort of adolescents in northern Sardinia, reported a seroprevalence of 6.1% [37], while a more recent case of neuroborreliosis diagnosed in a sedentary septuagenarian patient [40] has reignited concerns about the potential underdiagnosis and silent endemicity of LB in the region.

In light of these considerations, the present study aims to explore the seroprevalence of anti-*Borrelia* antibodies in Sardinia, Italy, with the dual objective of refining epidemiological knowledge and enabling the informed drafting of more effective and regionally tailored surveillance strategies.

## 2. Materials and Methods

### 2.1. Study Setting

This study is part of the project “Surveillance of the Presence of *Borrelia* Spirochetes in Different Arthropod Species in Sardinia and Investigation of a Population at Risk” carried out at the Experimental Zooprophylactic Institute of Sardinia (IZS) (Grunt RC IZS SA 04/19), funded by the Italian Ministry of Health in collaboration with the Department of Veterinary Public Health, Food Safety, and Collegial Health Protection Bodies of the Sardinia Region. A sero-prevalence study was conducted on serum samples from the serum bank of the Immunopathology Laboratory at the University Hospital of Sassari, collected between 2006 and 2014. The serum library consisted of serum samples sent to the laboratory based on a diagnostic hypothesis of rheumatic disease. Since Sardinia is considered a borreliosis-free area, none of the patients who did not present a specific risk, such as travel outside Sardinia, had been tested for borreliosis at the time of sampling. Therefore, for the purposes of this study, borreliosis remained, along with others, a possible diagnosis. Of all the samples stored, 58 were negative to the panel of 8 specific autoantibodies relevant for the differential diagnosis of rheumatic diseases. These samples were included in the study for the detection of antibodies to *Borrelia* spp.

Given the nature of the study and the clinical background of the patients, and considering that the serum samples derived from individuals with suspected rheumatic disease, a condition generally associated with late or persistent immune responses rather than early infection, IgG detection was considered the most appropriate approach for the screening on this cohort.

Therefore, the first level consisted in IgG detection by the indirect immunofluorescence assay (IFA) and the enzyme-linked immunosorbent assay (ELISA). Subsequently, a second level confirmatory test was performed by Western blot.

The study was approved by the Ethics Committee of Regional Health Agency of Sardinia—ARES Sardinia (Protocol No. 451/2022/CE, approved on 8 November 2022)—and all collected data were handled in compliance with current regulations and privacy protection standards.

### 2.2. The Indirect Immunofluorescence Assay (IFA)

An indirect immunofluorescence assay (IFA) was performed as a first-line screening test to detect IgG antibodies against *Borrelia burgdorferi* sensu stricto in human serum samples.

The analysis was conducted at the serology laboratory of the Department of Animal Health at the IZS of Sassari, using a commercial IFA kit provided by Fuller Laboratories (Fullerton, CA, USA) in accordance with the commercial instructions. In particular, serum samples, after proper dilution in PBS, were incubated at 37 °C for 30 min. In the end incubation were rinsed and incubated with secondary antibody Dynalight 488-coniugated. The slides were examined under a fluorescence microscope at 400× magnification. The intensity, distribution, and pattern of fluorescence were compared with those observed in the positive and negative control wells

### 2.3. The Enzyme-Linked Immunosorbent Assay (ELISA)

To increase the sensitivity of screening, ELISA as a first-level screening test analysis in addition to IFA testing was used [41,42]. This assay is a qualitative immunoenzymatic test designed to detect specific IgG antibodies against *B. burgdorferi* in human serum. The microplates used in the test are coated with specific antigens that selectively bind to the corresponding antibodies present in the sample. After an initial washing step to remove unbound materials, a horseradish peroxidase conjugated reagent is added, which binds to the captured antibodies. A subsequent washing step eliminates any unbound conjugate. The immune complex is then visualized by introducing Tetramethylbenzidine substrate, resulting in a blue reaction product. The intensity of this color is directly proportional to the quantity of specific antibodies in the sample. To terminate the reaction, sulfuric acid is added, producing a yellow endpoint color. The absorbance is then measured at 450/620 nm using an ELISA microplate reader to quantify the antibody levels.

The analysis was conducted by the staff of the Immunohematology and Transfusion Medicine Service the University Hospital of Sassari, in collaboration with IZS of Sardinia (Italy), using a kit from Demeditec (Demeditec Diagnostics GmbH; Kiel 24145, Germany).

### 2.4. Western Blot

The Western blot, or immunoblot, is a second-level confirmatory test with a diagnostic sensitivity of 100% and a specificity of 99.4%. A qualitative in vitro test was performed using a kit from Euroimmun (Euroimmun Italia; Padova 35127, Italy) on all samples that tested positive in the IFA and ELISA screening tests.

The EUROIMMUN kit is able to detect human IgM and IgG antibodies to *Borrelia* antigens in serum or plasma. In particular, the *Borrelia*-specific antigens detected are: p83, p58, p43, p39, p30, p21, OspC, DbpA/Osp17, p14, VlsE, p41. They represent the immunodominant antigens of the four *Borrelia* genospecies: *B. burgdorferi sensu stricto*, *Borrelia garinii*, *Borrelia afzelii*, *Borrelia spielmanii* and *Borrelia bavariensis* known to cause Lyme borreliosis. Immunoblot strips are incubated with diluted patient serum. If the samples are positive, specific IgG (and also IgM) antibodies bind to the corresponding antigenic site.

**Figure 1:**
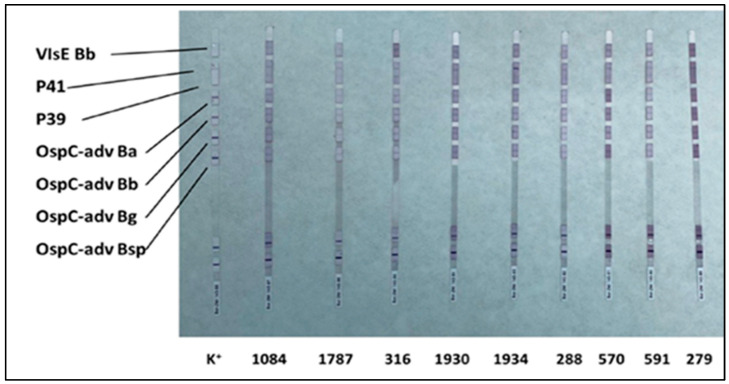
Western blot IgM strips for the nine ELISA-positive samples and control (K^+^). Bands corresponding to *Borrelia*-specific antigens are indicated.

**Figure 2:**
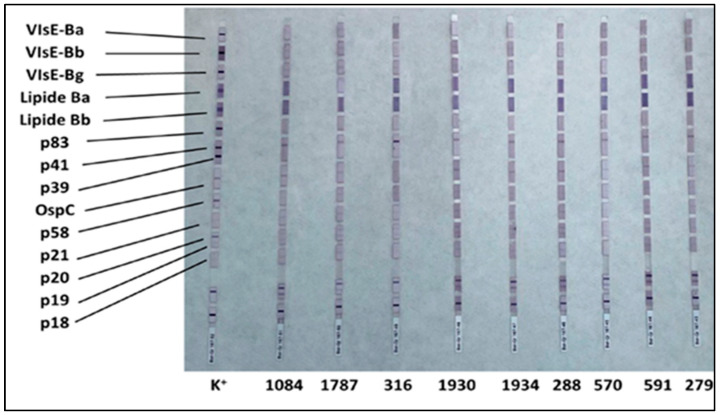
Western blot IgG profiles for all positive samples, including the reference positive control. Prominent bands for VlsE antigens are highlighted.

To visualize the bound antibodies, a second incubation is performed with human anti-IgG and anti-IgM antibodies conjugated with an enzyme (enzyme conjugate), which catalyzes a colorimetric reaction (violet color—Figure 1 and Figure 2), enabling identification of specific antibody binding.

## 3. Results

A total of 58 human serum samples were selected and analyzed through both IFA and ELISA to detect the presence of IgG antibodies against *Borrelia burgdorferi*.

Among the 58 human sera tested by IFA, seven samples were positive for IgG antibodies. Five samples showed strong fluorescence, and two were weakly positive. The 58 specimens were analyzed by ELISA, and 9 (15.5%) yielded positive results for IgG antibodies. All seven IFA-positive samples were included among the nine ELISA positives.

To validate seropositivity, Western blot analysis was performed to detect specific IgM and IgG antibodies. IgM profiling revealed the presence of multiple reactive bands across all nine ELISA-positive samples (Table 1; Figure 1).

The most frequently detected antibodies were those against OspC-adv of *Borrelia burgdorferi*, *Borrelia garinii*, and *Borrelia afzelii*. Among these, OspC-adv of *Borrelia afzelii* elicited the most pronounced immunoreactivity, exhibiting medium (++ intensity) bands in a majority of samples (Table 1).

The presence of IgG antibodies was also confirmed via Western blotting (Table 2; Figure 2). The most frequently detected antigens were VlsE proteins of *Borrelia garinii* and *Borrelia burgdorferi sensu stricto*, with the latter displaying the highest intensity across samples, thereby supporting the ELISA findings.

## 4. Discussion

Lyme borreliosis ranks among the most intricate Public Health concerns associated with arthropod-borne diseases, owing to its marked clinical polymorphism, insidious transmission dynamics, and the persistent challenges it poses as regards both diagnosis and surveillance [43,44,45].

The variability of surveillance systems across Europe, together with the lack of harmonized diagnostic criteria and the inconsistent use of serological assays, has made it difficult to gain a coherent and comparable epidemiological insight [7,16,23,24,43]. In this study, serological analysis of 58 human serum samples revealed IgG antibodies to *Borrelia burgdorferi* in 7 samples that tested positive by IFA and in 9 samples that tested positive by ELISA. Western blot analysis confirmed the presence of IgG antibodies and revealed positivity for IgM antibodies, with the most frequent reactivity observed against OspC-adv of *Borrelia burgdorferi*, *Borrelia garinii*, and *Borrelia afzelii*, and against the VlsE proteins of *Borrelia garinii* and *Borrelia burgdorferi sensu stricto*, respectively. These findings underscore the critical importance of strengthening surveillance activities across both community and healthcare settings, through diverse and complementary approaches [46,47,48].

Italy, and particularly its insular regions, remain largely unexplored from an epidemiological perspective, mainly due to the lack of systematic data collection [34,35,36,37,38,39,40]. In this context, the serological study conducted in Sardinia represents a key contribution to understanding the silent circulation of *Borrelia* spp. in a region where the perception of risk is still very limited and fragmented [36,37].

The detection of anti-*Borrelia* spp. antibodies in a Sardinian cohort originally investigated for rheumatic conditions, offers compelling, albeit preliminary, evidence of a potential silent endemicity of LB on the island, historically deemed peripheral in the Mediterranean tick-borne disease landscape [37]. This finding, although derived from a descriptive analysis and a relatively small sample, provides further serological evidence over two decades that *Borrelia* spp. may be circulating more extensively in Sardinia than previously assumed.

While the antibody data should be interpreted with caution, its indicative value cannot be ignored. Indeed, seropositivity is not necessarily synonymous with active disease, although it constitutes an immunological trace of previous exposure to the pathogen [16,49]. Moreover, in low-surveillance contexts, this indicator takes on an amplified meaning, acting as a sentinel signal of circulation which, while it may be silent, is not devoid of health impact [7,36,45,49,50,51]. For this very reason, the high prevalence observed deserves further investigation in prospective studies. Such research should aim to be multicentered and supported by molecular confirmation or cultures, where applicable.

The three-pronged diagnostic approach based on IFA, ELISA and Western blot, yielded a consistent immunoreactive profile across multiple *Borrelia* antigens, notably those of *Borrelia afzelii*, *Borrelia garinii*, and *Borrelia burgdorferi sensu stricto*.

In this regard, our study, although based on a limited number of samples, confirms a higher sensitivity of IFA than ELISA [41,42].

The frequent detection of OspC and VlsE, distinctive virulence factors involved in immune evasion and early pathogenesis, not only validates the robustness of the serological response observed but also aligns with European epidemiological trends in which *Borrelia afzelii* predominates in cutaneous forms and *Borrelia garinii* in neuroborreliosis [1,2,3]. It should be noted that protein-based assays have limited reliability for definitive genospecies assignment, as cross-reactivity and individual immune variability may affect specificity; molecular methods such as PCR remain the gold standard. Nevertheless, this study revealed for the first time this molecular pattern previously uncategorized in Sardinia, mirrors antigenic signatures documented in Central and Eastern Europe, suggesting either shared ecological drivers or migratory connectivity through avian hosts [24,52,53,54].

In the absence of a structured surveillance system, LB remains an elusive entity in Sardinia. This scenario reflects a broader dilemma in Public Health, where diagnostic inattention and systemic underreporting contribute to an artificially obscured epidemiological picture. The sporadicity of documented clinical cases, juxtaposed with entomological evidence of *Ixodes ricinus* on the island [4,5,6], brings to mind the “iceberg phenomenon” often cited in zoonotic disease epidemiology, wherein only the most overt cases surface above the diagnostic threshold [26,36].

The sera analysed in this study were obtained from hospitalised patients initially investigated for rheumatic disease and who therefore represent a limited segment of the clinical spectrum [55]. Nonetheless, the detection of nine positive samples among those tested provides meaningful evidence of *Borrelia* spp. exposure in this population. This observation supports the hypothesis that Lyme borreliosis is under-recognised and under-reported in Sardinia and highlights the need for systematic and standardised surveillance to define its actual epidemiological impact.

Furthermore, the use of sera originally collected for rheumatologic assessment highlights a possible diagnostic overlap between LB and autoimmune conditions, which may contribute to under-recognition of the disease, particularly in non-endemic areas with limited clinical awareness [7,8,9].

Given the constraints on healthcare and surveillance resources, future investigations should prioritise approaches that can provide reliable population-level estimates in a cost-effective manner. Population-based seroprevalence studies, widely used to estimate Lyme borreliosis incidence in other European settings, offer a pragmatic and complementary alternative to prospective incidence studies because they are less complex, less costly, and can be completed within quickly timeframes [18,41,42,43].

From a One Health perspective, Sardinia presents the ecological conditions required to sustain an endemic *Borrelia* spp. cycle. The region includes wooded and pasture areas, a high density of wild and domestic reservoirs, a workforce involved in forestry and agriculture, and a recent increase in deer populations, which may act as reservoirs [44,56,57]. Recent climate trends with warmer winters and higher humidity are likely reshaping tick phenology and extending periods of vector-host interaction [11,12,13,44]. These observations underscore that even insular Mediterranean ecosystems, traditionally considered less favorable for tick-borne pathogens, may support the persistence and circulation of *Borrelia* spp., particularly under the influence of ongoing environmental and anthropogenic changes [36,56]. On a national scale, Italy’s fragmented surveillance framework for LB, currently reliant on passive case reporting without standardized serological mapping, presents a major obstacle to both intra- and interregional comparability. The northern regions, particularly Trentino-Alto Adige and Friuli Venezia Giulia, have established diagnostic pathways and integrated vector monitoring protocols that are virtually absent in the South and on the islands [26,31,32,58,59,60]. The stark asymmetry in case reporting between Alpine and Mediterranean areas, while partially attributable to climatic gradients, likely reflects a deeper structural gap in Public Health preparedness and awareness. In this light, it is essential to interpret our findings not only as a call for increased diagnostic vigilance but also as an initial step toward the development of an active, region-specific surveillance system [34,35,36,37]. Integrating serological screening into broader Public Health initiatives, such as occupational health programs targeting forestry and agricultural workers, could unmask latent endemicity and shape targeted interventions.

At the international level, our data resonate with the growing recognition that LB is currently underdiagnosed in southern Europe, despite the confirmed presence of competent vectors and reservoirs [14,15,16,17]. The European Centre for Disease Prevention and Control (ECDC) has repeatedly emphasized the need for harmonized surveillance across member states, citing discrepancies in diagnostic criteria and case definitions as major barriers to understanding the true burden of disease [61].

In this context, Sardinia represents a blind spot but also an opportunity: a geographic niche where coordinated research could yield insights with broader applicability to other “low-reporting” Mediterranean regions.

## 5. Conclusions

The present study reopens the epidemiological file on *Borrelia* spp. in Sardinia, challenging prevailing assumptions regarding the southern geographical limits of LB in Europe. Sardinia should not be considered a marginal context within the European epidemiological landscape. Instead, it represents a potential emerging hotspot of strategic relevance for the broader Mediterranean basin. Its geographical position, ecological features, and vector biodiversity position it as a sentinel territory, well-suited to piloting integrated surveillance models within the One Health framework.

From this perspective, Sardinia offers an optimal testing ground for implementing integrated systems that combine entomological, clinical, and sero-epidemiological surveillance. Although the study has limitations, including the relatively small sample size, the absence of PCR-based confirmation, and the fact that the serum library did not contain samples beyond 2014, the findings nonetheless provide meaningful evidence of *Borrelia* exposure and support the notion that LB may be underreported in Sardinia. The findings underscore the urgent need for systematic sero-epidemiological studies, comprehensive vector mapping, and structured public health strategies. These efforts are essential to support coherent and equitable epidemiological surveillance pathways and to address the diagnostic gap between northern and southern regions. A centralized regional database is therefore critically needed. The systematic recording of tick bites, confirmed LB diagnoses, and serological results would allow for real-time monitoring of epidemiological trends, including within susceptible cohorts such as forestry and public land workers.

Such a tool would enhance preparedness and support timely, data-driven public health responses, particularly in the context of ongoing climatic and ecological changes.

## Figures and Tables

**Table 1:** Western blot results for IgM antibodies. Band intensity is denoted as follows: + = weak; ++ = medium; +++ = strong.

	Antigen IgM						
ID	VIsE Bb	p41	p39	OspC-adv Ba	OspC-adv Bb	OspC-adv Bg	OspC-adv Bsp
279	++	++	-	+	+	+	+
591	+	+	-	+	+	+	+
570	++	++	-	++	++	++	++
288	+	+	-	+	+	+	+
1934	++	+++	-	++	+	+	+
1930	++	++	-	+	+	+	+
316	++	+	+	++	++	++	++
1787	+	+	-	+	+	+	+
1084	+	+	+	+	+	+	+

**Table 2:** Western blot results for IgG antibodies. Band intensity: + = weak; ++ = medium; +++ = strong.

	Antigen IgG													
ID	VIsE Ba	VIsE Bb	VIsE Bg	Lipide Ba	Lipide Bb	p83	p41	p39	OspC	p58	p21	p20	p19	p18
279	+	+	+	-	-	-	++	-	++	+	+	+	+	+
591	+	++	-	-	-	-	++	-	+++	+	+	+	+	+
570	+	-	-	-	-	-	++	-	-	-	-	-	-	-
288	-	+	+	-	-	-	++	-	++	-	+	++	++	+
1934	-	+	+	-	-	-	++	-	++	-	-	+	+	+
1930	-	++	+	-	-	-	++	-	++	+	+	+	+	+
316	+	-	+	-	-	-	+++	-	+++	-	+	+	+	-
1787	+	+	+	-	-	-	++	-	++	+	+	+	+	+
1084	-	+	+	-	-	-	++	-	++	-	+	+	+	+

## Data Availability

The data presented in this study are available on reasonable request from the corresponding author. The data are not publicly available due to privacy and ethical restrictions.
